# Knowledge of, Attitudes towards, and Practices of Intranasal Corticosteroids Usage among the Allergic Rhinitis Patients of Northern Saudi Arabia: A Cross-Sectional Study

**DOI:** 10.3390/healthcare11040537

**Published:** 2023-02-11

**Authors:** Abdullah N. Al-Rasheedi

**Affiliations:** Department of Otolaryngology and Head and Neck Surgery, College of Medicine, Jouf University, Sakaka 72388, Saudi Arabia; analrashedi@ju.edu.sa; Tel.: +966-599739619

**Keywords:** allergic rhinitis, smoking, knowledge, adherence, attitude, Saudi Arabia

## Abstract

Intranasal corticosteroids (INCS) are generally safe and effective treatments for allergic rhinitis (AR). The improper use of INCS may not alleviate AR symptoms, and it could lead to complications and an impaired quality of life. We evaluated the knowledge of, attitudes towards, and practices of INCS usage and associated factors among AR patients using a pretested Arabic questionnaire. Of the 400 participating AR patients, 39.3%, 29.0%, and 36.5% had poor scores for knowledge, attitude, and practice, respectively. We found a significant association between knowledge and education (*p* < 0.001) and follow-up facilities (*p* = 0.036). The attitude category was significantly associated with age (*p* = 0.003), marital status (*p* = 0.004), and type of allergic patients (*p* < 0.001), and the practice category was significantly associated with education (*p* = 0.027), type of allergic patients (*p* = 0.008), and follow-up facilities (*p* = 0.030). Smoking status was significantly associated with all three categories. Furthermore, we found a positive correlation between knowledge and practice scores (Spearman’s rho of 0.451, *p* < 0.001). We recommend improving AR patients’ knowledge of the proper practices of INCS through health education programs. Furthermore, we recommend an exploratory mixed-method survey on the INCS usage among AR patients that involves other provinces in the KSA.

## 1. Introduction

Allergic rhinitis (AR) is an atopic disease characterized by sneezing, runny nose, itchy nose, nasal congestion, and post-nasal drip [1,2]. Even though it is difficult to measure the exact prevalence of AR, it is considered one of the most common diseases worldwide [3,4]. Recent epidemiological surveys in the kingdom of Saudi Arabia (KSA) have indicated that AR’s prevalence ranges from 15 to 40%, depending on age, gender, and other sociodemographic characteristics [5,6]. AR is a long-standing disease commonly associated with a spectrum of other allergic comorbid conditions, namely, bronchial asthma, blocked eustachian tubes, food allergies, turbinate hypertrophy, pharyngitis, laryngitis, and conjunctivitis [7,8]. AR can significantly impair the quality of life of an affected adult due to its negative effect on all dimensions of sleep, work, academic performance, mood, and daily activities [9,10,11]. Several researchers have reported that both acute and chronic AR affect adults and children [12]. The European Position Paper on Rhinosinusitis and Nasal Polyps (EPOS) published in 2020 found that there was a significant reduction in the quality of life of affected children [13]. Another study by Chmielik et al. reported decreased well-being, discomfort, and low perceptions of health in children with chronic rhinitis [14].

The treatments for AR include antihistamines, nasal decongestants, intranasal corticosteroids (INCS), cromoglicic acid (nasalcrom), leukotriene receptor blockers, avoidance of allergens, and nasal anti-cholinergic, immunotherapy, or combination therapy [1,15]. The British Society for Allergy and Clinical Immunology (BSACI) and the American Academy of Allergy Asthma and Immunology have suggested initiating INCS alone for mild to moderate AR patients [16,17]. Numerous researchers have evaluated the safety of INCS in the past, and it is generally considered to be safe and effective, with few side effects [18,19]. The control of any health issues, including AR, ultimately depends upon a patient’s adherence to usage and follow-up regarding the treating physician’s advice. Failure to practice medication adherence may lead to treatment failure and be detrimental to the healthcare system [20,21]. Improper and inadequate use of INCS treatment may not alleviate AR symptoms and may lead to several complications and an impaired quality of life [22,23]. A study conducted by Rajasekaran et al. reported that AR patients’ knowledge was low regarding AR symptoms and treatment. Most of their study participants had poor attitudes and were concerned about the long-term side effects and their dependence on the medications prescribed for managing AR symptoms [24]. When evaluating the medication adherence practices of AR patients using INCS, Majnith et al. found that only 58.9% of their study’s participants adhered to the INCS treatments as prescribed [25]. On assessing AR patients’ attitudes toward INCS treatment (mometasone furoate), Fromer et al. stated that most of their participants found that mometasone furoate nasal spray was easy to use and administer [26]. In spite of the significant decrease in quality of life expressed the participants in a study by Katelaris et al., 44% had never used INCS or had not used INCS in the past year. Furthermore, their survey respondents’ knowledge regarding INCS and its usage was poor [27]. Patients’ and prescribers’ related factors may contribute to poor practices in the usage of INCS. A study conducted by Ocak et al. among Turkish adult AR patients stated that poor adherence practices in using INCS was higher among patients with lesser education [21]. Considering the increased availability of over the counter INCS products at pharmacies, Bridgeman et al. stated that community pharmacists’ knowledge and the usage practices of AR patients are also important factors in optimal knowledge and practice [23].

In the KSA, AR patients are diagnosed and treated by ENT specialists at specialist hospitals, as well as at primary health centers by primary care physicians. Continuous and accurate assessment of knowledge, attitudes, and practices towards INCS usage is essential for planning care services for AR patients. However, limited data are available on this subject, especially in the Aljouf region of the KSA. Considering the region-specific required data, the present study assessed the knowledge, attitudes, and practices towards INCS usage and associated factors among AR patients in the northern KSA to find correlations between knowledge, attitude, and practice scores.

## 2. Materials and Methods

### 2.1. Study Description and Sampling Strategy

The present analytical cross-sectional study was conducted from June to November 2022. It included the AR patients registered in the ENT clinics of specialist hospitals and primary health centers in the Aljouf region. This region is situated in the northern part of the Kingdom of Saudi Arabia (KSA) and has a population of 500,000. There are 75 primary health centers and 4 specialist hospitals in this region. This study’s sample size was estimated using the Raosoft online sample size calculator. The Raosoft online calculator uses Cochran’s formula (*n* = z^2^pq/e^2^) for estimating sample size. The following values were considered while calculating the sample size: *n* = minimum required sample size, z = 1.96 at s confidence level of 95%, *p* = expected proportion of 50%, q = 1 − *p*, and e = margin of error at 5%. Considering all the specified values, the minimum required sample size was 377, and it was rounded to 400. A consecutive sampling method was applied to select the study participants (the AR patients). In this method, all the registered AR patients were selected. We began with the newest registered and follow-up patients to the last until the required sample size was achieved.

### 2.2. Inclusion and Exclusion Criteria

Of the available 75 primary health centers and 4 specialist hospitals, we randomly selected 10 health centers and 2 hospitals. The present study included all of the diagnosed and registered AR patients who could read and write in Arabic and had been on INCS for a minimum period of one month. We excluded the AR patients who were under 18 years of age, using other treatment methods (such as antihistamines), and unwilling to participate in the survey. Furthermore, we excluded the AR patients who had registered for the first time at the selected healthcare facilities. We invited every fifth patient during their follow-up visit during the data collection period.

### 2.3. Data Collection Procedure

After obtaining ethical clearance (Qurrayat Health Affairs, Saudi Arabia and Approval number 127) and the necessary permissions from the Ministry of Health, Jouf Affairs, the data collectors in the present study began the data collection by using a self-administered, validated Arabic version of the knowledge, attitude, and practice–intranasal corticosteroid questionnaire (KAP-INCS). This questionnaire was adapted from an open-source, valid, and reliable tool used to evaluate an AR patient’s KAP regarding INCS treatment [28]. We followed the standard protocols for translating the questionnaire into Arabic [29,30]. Firstly, a panel of experts from ENT, family medicine, and the pharmacy department verified the content through a group discussion. Secondly, two bilingual (English and Arabic language) experts translated the English version into Arabic. In the next stage, another set of non-medical bilingual experts back-translated the KAP-INCS questionnaire into English. The team involved acknowledged that the back-translated version retained the original meaning of the KAP-INCS questionnaire. Finally, we performed a pilot survey among 30 AR patients for local adaptability. All pilot study AR patients expressed that the Arabic version of the KAP-INCS questionnaire was clear, and they found it easy to respond to the items. On average, the participants took 10 min to complete the survey. Furthermore, Cronbach’s alpha values for the Arabic version of the questionnaire were 0.83 (knowledge), 0.76 (attitude), and 0.84 (practice). This questionnaire consisted of two parts. The first part inquired about the sociodemographic status of the study population. The second part consisted of the KAP related to INCS use. Each section of the KAP consisted of four questions. The AR patients responded yes, not sure, or no in the knowledge section. This knowledge section inquired about the AR patients’ comprehension regarding side effects, importance, and correct ways of using INCS. We scored 2 for yes, 1 for not sure, and 0 for no in the knowledge section. The attitude section answers were given six ordered scores (5 to 0) for the responses of totally agree to totally disagree. In this section, AR patients responded with their agreement on their attitudes about knowing more about AR, their priorities regarding illness, and practices with treatment. Finally, the AR patients chose a response in the practice section from five possible choices: “always to almost never”, scored from 4 to 0. In the practice section, we evaluated the participants’ adherence to the prescribed INCS treatment and their follow-ups with their doctors as recommended. We computed the total scores for each section and categorized them into poor (less than 60% of the total score), average (60 to 80% of the total score), and excellent (more than 80% of the total score). Furthermore, we grouped together low and average scores to compare with the excellent scores, per Bloom’s criteria. A score of less than 80% for each KAP category was considered suboptimal.

### 2.4. Statistical Analysis

The statistical package for social sciences (SPSS) version 21.0 was used for exporting, coding, and analyzing the data. We depicted the descriptive analysis as frequencies and proportions (*n*, %). The data were tested for normality assumptions, and we applied Spearman’s rank correlation test to find the correlations between the KAP scores. Furthermore, we performed the chi-square test to find the relationships between the sociodemographic characteristics and the KAP categories. A *p*-value of less than 0.05 was set as a statistically significant value for all two-tailed statistical tests.

## 3. Results

Of the 400 AR patients who responded who were on INCS, the majority (52.7%) of them were females, 25 to 40 years old (39.5%), married (58%), educated at the university level and above (60.7%), and non-smokers (79.3%). Regarding AR status, nearly one-third (34.7%) of the patients were suffering from mild or intermittent AR, and most of them (49.3%) were receiving treatment at a specialty hospital (Table 1).

**Figure 1:**
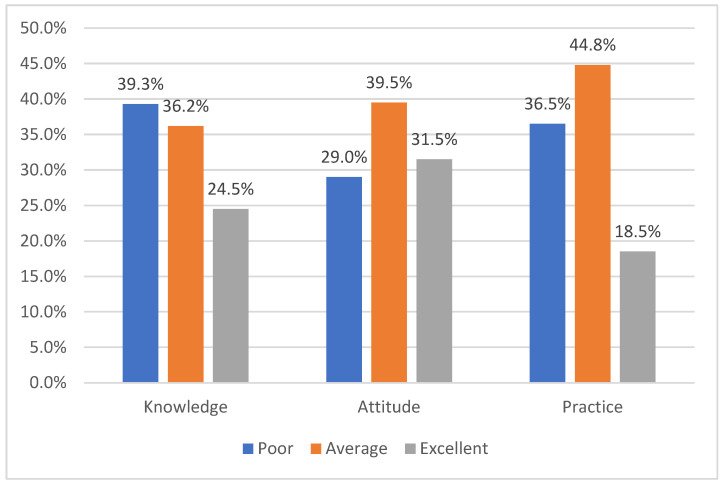
KAP categories for INCS usage among the AR patients (*n* = 400).

Figure 1 depicts the KAP categories for INCS usage among the AR patients. Of all the participating AR patients, 39.3% had a poor score for knowledge, 29.0% had a poor score for attitude, and 36.5% had a poor score for the practice category, and 24.5%, 31.5%, and 18.5% had excellent scores for the knowledge, attitude, and practice categories, respectively.

Regarding knowledge related to INCS, significant associations are found with occupation (*p* = 0.004), education (*p* < 0.001), smoking status (*p* = 0.006), and follow-up facilities (*p* = 0.036). The attitude category is significantly associated with age (*p* = 0.003), marital status (*p* = 0.004), smoking status (*p* = 0.006), and type of allergic patient (*p* < 0.001), and the practice category is significantly associated with current married status (*p* = 0.029), educational qualification (*p* = 0.027), smoking status (*p* = 0.038), type of allergic patient (*p* = 0.008), and follow-up facilities (*p* = 0.030) (Table 2).

Table 3 depicts the correlations between the KAP scores for INCS among the AR patients. We found positive correlations between knowledge and attitude (rho = 0.153, *p* = 0.015), knowledge and practice (rho = 0.451, *p* < 0.001), and attitude and practice (rho = 0.297, *p* = 0.003).

## 4. Discussion

The present study assessed the knowledge, attitudes, and practices regarding INCS treatment among AR patients of the Aljouf region of northern Saudi Arabia. Of the 400 studied AR patients, 34.7% suffered from mild forms of AR. The present study’s participant characteristics were similar to a nationwide study conducted by Almehezia et al. in 2019. In their research, nearly one-third of the patients presented with milder forms of AR, and a significantly higher proportion of the patients that presented milder forms of AR were female [31].

Sufficient knowledge of the disease and its management are essential for patients suffering from a chronic disease, and poor knowledge can lead to improper adherence to the prescribed treatments and their associated complications [21,32]. The present study found that only 24.5% of the AR patients had sufficient knowledge about the usage of INCS, and the remaining participants’ knowledge was suboptimal. A single-center study conducted in the Riyadh region of the KSA by Almutairi et al. stated that more than 70% of their participants had knowledge of the benefits of INCS. Nonetheless, only approximately half of the participants had knowledge about the techniques for using nasal steroids [33]. The possible difference between our research and that of Almutairi et al. may be the different data collection tools used to evaluate the AR patients’ knowledge about INCS. We used the newly developed KAP-INCS, but the study by Almutairi et al. assessed patient knowledge with the benefits and safety concerns related to treatment. The current study’s results indicated that focused efforts are needed by the concerned healthcare managers to increase AR patients’ awareness of and knowledge about INCS. Other studies conducted in the USA and Turkey reported similar findings. They also reported that the knowledge gap among AR patients could be due to several factors related to patients and physicians [34,35]. The participating AR patients’ knowledge about the usage of INCS was significantly associated with their education (*p* < 0.001), smoking status (*p* = 0.007), and follow-up facilities (*p* = 0.036). Similarly, a recent study on INCS usage found a significant association between smoking status and knowledge [33]. It is worth mentioning that smoking status is one of the risk factors for AR, and it may reduce the effectiveness of steroids by reducing their sensitivity [31,36]. Furthermore, the present study explored a positive association between education status and the knowledge category. Hence, a patient’s education is also a critical factor to consider when a physician imparts INCS knowledge, as the instructions in the medication leaflet may not be understandable by all AR patients [21,37]. Our findings are supported by those of Lee et al., who concluded that the patient must understand the instructions properly for better and maximum utilization of INCS treatment [37].

Patients’ attitudes and beliefs are critical for compliance with prescribed INCS treatment and other healthcare services [23,26,38]. We found that only approximately one-third of AR patients had excellent attitudes towards INCS, and the rest of the patients had either average or poor attitudes. The low level of attitude among AR patients indicates a need for improved awareness and a positive attitude. This can essentially be completed through treating physicians and healthcare providers. In contrast to the present study, a higher proportion of favorable attitudes was noted in a study by Abdullah et al. [39]. The possible variation in the findings may be due to the inclusion of the study participants. The present study invited the general population, and the study by Abdullah et al. was conducted among primary care physicians. The present study found a significantly poor attitude among the mild and intermittent AR patients (*p* < 0.001) and smokers (*p* = 0.006). Firstly, the poor attitude toward INCS treatment among the participants could be due to the improvement in symptoms after the initial dosage among mild cases and non-adherence to the prescribed regimen. The present survey’s findings are supported by the findings of Manjit et al. (2022). Their survey found that the absence of symptoms and taking medications intermittently were significant factors for non-adherence [25].

Similar to knowledge and attitude, we found that a low proportion (18.5%) of AR patients had excellent practices with INCS treatment for their AR. In contrast, a recent survey by Mohammed et al. in 2022 showed that only approximately 5% of the participants had a poor level of practice [40]. The striking dissimilarities are due to the study tools used and the participants involved in the survey. The study by Mohammed et al. assessed the practices of community pharmacists in their general management of AR. It was expected that healthcare workers’ practices regarding adherence would be high, as their health literacy is higher than a patient’s. The practice categories were significantly associated with education, smoking, type of AR patient, and follow-up facilities. We found that poor practices were considerably higher among the patients who received over the counter INCS treatments at a pharmacy (*p* = 0.03). The possible factors for the poor practices among smokers could be less sensitization and effectiveness of steroids compared to non-smokers [41]. Similar to our findings, Ocak et al. found a significant association with educational qualification [21]. It was proven that poor adherence practices among mild and intermittent cases is a common phenomenon [25]. Furthermore, the significantly poor practices among the AR patients following up at a pharmacy could be due to a lack of sufficient knowledge imparted by the pharmacist dispensing the INCS. Our findings support the theory that evaluating the factors associated with non-adherence to INCS can help policymakers address constraints faced by AR patients and eventually improve adherence practices and clinical outcomes. Finally, we found a significant positive correlation between knowledge and the attitude and practice scores. Similar to the current findings, Mohammed et al. found positive correlations between knowledge and attitude (*p* = 0.000), attitude and practice (*p* = 0.009), and knowledge and practice (*p* = 0.000) [40]. The similar results between the studies are due to disease knowledge and its direct association with patient adherence to health protocols. This indicates that improving a patient’s knowledge about INCS usage is essential to managing AR patients.

The researchers used the best possible and systematic methods to conduct the present research. Nonetheless, a few limitations must addressed. Firstly, as an important limitation of the cross-sectional study method that concurrently evaluated the risk factors and outcomes, we could not identify the direction of either association or causation. Secondly, self-reported and recall biases cannot be ignored as the current survey is questionnaire-based. Thirdly, the present study was conducted in healthcare facilities; thus, the results of the study cannot be generalized to the general population. Fourthly, the present study was limited to the northern region of the KSA. Considering the vast differences in sociocultural characteristics among different regions of the KSA, the present study’s findings may not be generalized to all provinces of the KSA and middle eastern countries.

## 5. Conclusions

The present study explored the suboptimal knowledge, attitudes, and practices of INCS usage among AR patients. Smoking, education level, milder forms of AR, and follow-up facilities were the significant factors associated with the KAP categories. A positive correlation between the knowledge scores and the attitudes and practices indicated that improving a patient’s knowledge of INCS usage is essential to managing AR patients. Furthermore, we noted that poor practices were significantly higher among the AR patients receiving follow-up INCS treatment using over the counter products from a pharmacy. Hence, we recommend improving the knowledge of AR patients through awareness-raising programs that are primarily target-oriented. Furthermore, community pharmacists require that a focused training program on the protocols be followed when distributing INCS treatments. Finally, we recommend an exploratory prospective mixed-method KAP study to evaluate the qualitative components involving the other provinces of the KSA.

## Figures and Tables

**Table 1:** Sociodemographic characteristics of the AR patients (*n* = 400).

Variables	Frequency	Proportion
Age (years):		
Less than 25 years	135	33.8
25 to 40 years	158	39.5
Above 40 years	107	26.7
Gender:		
Male	186	46.5
Female	214	53.5
Marital status:		
Married	232	58.0
Single	137	34.3
Divorced/widowed	31	7.7
Occupation:		
Government	161	40.3
Private	105	26.2
Self-employed/business	64	16.0
Unemployed	70	17.5
Education:		
Up to high school	157	39.3
University level	243	60.7
Income:		
Less than 5000 SAR	94	23.5
5000 to 7000 SAR	185	46.3
More than 7000 SAR	121	30.2
Residence:		
City/urban	315	78.8
Village/rural	85	21.2
Smoking status:		
Yes	83	20.7
No	317	79.3
Allergic rhinitis status:		
Mild intermittent	139	34.7
Mild regular	111	27.8
Moderate to severe intermittent	92	23.0
Moderate to severe permanent	58	14.5
Where are you receiving treatment for your allergic rhinitis management?		
Specialist at hospital	197	49.3
Primary health center	135	33.7
Over the counter drugs at a pharmacy	68	17.0
Duration of INCS usage		
Less than 1 year	99	24.8
1 to 3 years	162	40.5
More than 3 years	139	34.7

**Table 2:** Relationship between each sociodemographic characteristic and each KAP category (*n* = 400).

Variables		Knowledge			Attitude			Practice		
	Total	Poor and Average (*n* = 302)	Excellent (*n* = 98)	*p*-Value	Poor and Average (*n* = 274)	Excellent (*n* = 126)	*p*-Value	Poor and Average (*n* = 325)	Excellent (*n* = 75)	*p*-Value
Age (years):				0.807			0.003 *			0.952
Less than 25 years	135	102	33		81	54		109	26	
25 to 40 years	158	117	41		107	51		128	30	
Above 40 years	107	83	24		86	21		88	19	
Gender:				0.287			0.908			0.910
Male	186	145	41		130	59		154	35	
Female	214	157	57		144	67		171	40	
Marital status:				0.392			0.004 *			0.029 *
Married	232	170	62		144	88		179	53	
Single	137	109	28		108	29		121	16	
Divorced/widowed	31	23	8		22	9		25	6	
Occupation:				0.004 *			0.314			0.382
Government	161	116	45		110	51		126	35	
Private	105	71	34		67	38		84	21	
Self-employed/business	64	53	11		43	21		54	10	
Unemployed	70	62	8		54	16		61	9	
Education:				<0.001 *			0.735			0.027 *
Up to high school	157	136	21		106	51		139	18	
University level	243	166	77		168	75		186	57	
Income:				0.098			0.043 *			0.174
Less than 5000 SAR	94	71	23		58	36		75	19	
5000 to 7000 SAR	185	139	46		123	62		151	34	
More than 7000 SAR	121	92	29		93	28		99	22	
Residence:				0.537			0.640			0.057
City/urban	315	240	75		214	101		262	53	
Village/rural	85	62	23		60	25		63	22	
Smoking status:				0.006 *			0.006 *			0.038 *
Yes	83	72	11		67	16		74	9	
No	317	230	87		207	110		251	66	
Allergic rhinitis status:				0.113			<0.001 *			0.008 *
Mild intermittent	139	107	32		111	28		118	21	
Mild regular	111	84	27		80	31		95	16	
Moderate to severe intermittent	92	67	25		52	40		82	10	
Moderate to severe permanent	58	44	14		31	27		40	18	
Follow up:				0.036 *			0.081			0.030 *
Specialist at hospital	197	148	49		134	63		156	41	
Primary health center	135	95	40		95	40		106	29	
Over the counter drugs at pharmacy	68	59	9		45	23		63	5	

* Denotes significant value identified by chi-square test.

**Table 3:** Correlation between knowledge, attitude, and practice scores.

	Spearman’s Correlation Value (rho)	*p*-Value
Knowledge–attitude	0.153	0.015 *
Knowledge–practice	0.451	<0.001 *
Attitude–practice	0.297	0.003 *

* Denotes a significant correlation identified by the Spearman’s correlation test.

## Data Availability

The raw data used for analysis will be provided by the corresponding author on request.
